# Accuracy of genomic prediction using multiple Atlantic salmon populations

**DOI:** 10.1186/s12711-024-00907-5

**Published:** 2024-05-15

**Authors:** Afees A. Ajasa, Solomon A. Boison, Hans M. Gjøen, Marie Lillehammer

**Affiliations:** 1 Nofima (Norwegian Institute of Food, Fisheries and Aquaculture Research), PO Box 210, 1431 Ås, Norway; 2 Department of Animal and Aquacultural Sciences, Norwegian University of Life Sciences, 1430 Ås, Norway; 3 Mowi Genetics AS, Sandviksboder 77AB, Bergen, Norway

## Abstract

**Background:**

The accuracy of genomic prediction is partly determined by the size of the reference population. In Atlantic salmon breeding programs, four parallel populations often exist, thus offering the opportunity to increase the size of the reference set by combining these populations. By allowing a reduction in the number of records per population, multi-population prediction can potentially reduce cost and welfare issues related to the recording of traits, particularly for diseases. In this study, we evaluated the accuracy of multi- and across-population prediction of breeding values for resistance to amoebic gill disease (AGD) using all single nucleotide polymorphisms (SNPs) on a 55K chip or a selected subset of SNPs based on the signs of allele substitution effect estimates across populations, using both linear and nonlinear genomic prediction (GP) models in Atlantic salmon populations. In addition, we investigated genetic distance, genetic correlation estimated based on genomic relationships, and persistency of linkage disequilibrium (LD) phase across these populations.

**Results:**

The genetic distance between populations ranged from 0.03 to 0.07, while the genetic correlation ranged from 0.19 to 0.99. Nonetheless, compared to within-population prediction, there was limited or no impact of combining populations for multi-population prediction across the various models used or when using the selected subset of SNPs. The estimates of across-population prediction accuracy were low and to some extent proportional to the genetic correlation estimates. The persistency of LD phase between adjacent markers across populations using all SNP data ranged from 0.51 to 0.65, indicating that LD is poorly conserved across the studied populations.

**Conclusions:**

Our results show that a high genetic correlation and a high genetic relationship between populations do not guarantee a higher prediction accuracy from multi-population genomic prediction in Atlantic salmon.

**Supplementary Information:**

The online version contains supplementary material available at 10.1186/s12711-024-00907-5.

## Background

Genomic prediction (GP) is a form of marker assisted selection that relies on the presence of linkage disequilibrium (LD) between markers and quantitative trait loci (QTL). Its accuracy is in part determined by the size of the reference population [1]. In Atlantic salmon breeding programs, the generation interval is usually three to four years, and three to four parallel breeding populations (with a year between them) are usually maintained at any point in time in order to make seed stock available to the salmon industry each year (see review by Gjedrem [2]). Thus, a logical strategy to increase the size of the reference population and potentially the accuracy of GP is to combine these populations in one reference set (hereafter referred to as *multi-population prediction*). There might also be an interest to use marker effects estimated from one population to predict the breeding values of another population (hereafter referred to as *across-population prediction*) in order to reduce the need for phenotyping every year, particularly for disease resistance traits, which are expensive to record and have negative consequences for fish welfare. The challenge with both these strategies is that LD between markers and QTL might not be the same across populations [3], and more importantly, the LD phase between markers and QTL may differ between populations, particularly when low density markers are used or if it has been a long time since the populations diverged [4]. In addition, QTL effects might differ across populations [3] or different QTL may be segregating in each population [1]. Hence, accurate prediction of additive genetic effects might not be achieved with a large reference set that comprises multiple populations, and low across-population prediction accuracies might be obtained. Nonetheless, different approaches, such as increasing marker density [3], using nonlinear models [5], using carefully selected single nucleotide polymorphisms (SNPs) [6, 7] and others (see review by Lund et al. [5]) have been proposed to deal with some of these problems, with some success, mainly in cattle populations. Hence, given that the cost of genotyping is still a limiting factor, the use of nonlinear models and/or carefully selected SNPs need to be tested in aquaculture populations.

The objective of this study was to (i) evaluate the impact on accuracy of multi- and across-population prediction of breeding values for resistance to amoebic gill disease (AGD) by using all SNPs on a 55K chip or a selected subset of SNPs (based on the signs of allele substitution effect estimates across populations) using linear and nonlinear GP models, and (ii) estimate the persistency of LD phase between markers across different populations of Atlantic salmon.

## Methods

### Phenotypes

The dataset used in this study is fully described in Ajasa et al. [8]. Briefly, the study populations originate from a common base population of wild Atlantic salmon that were collected from two rivers off the west coast of Norway in the late 1960s and early 1970s [9]. Four parallel populations (with a year between them) were formed at the inception of the breeding program due to the generation interval of 4 years of Atlantic salmon, so that the needed seed stock is available for the industry each year. Initially, systematic mixing of these populations occurred in order to limit the amount of inbreeding in the breeding population. Eggs from these populations were exported to Ireland in the 1980s to form the Mowi Fanad strains [10]. The systematic mixing of the Norwegian populations stopped to a large extent in the first years of this century when DNA typing and the best linear unbiased prediction (BLUP) methodology were implemented. The populations used in this study are mainly from Mowi’s Norwegian nucleus populations, however, for the year classes (YC: year-class indicates the year the populations were taken to sea) 2015 and 2016, the Mowi’s Fanad populations were introgressed into the Mowi Norwegian populations. To distinguish the Norwegian and Irish (Fanad) populations of the YC 2015 and 2016, N and F, were appended to the names, respectively. In total, six populations (YC2015N, YC2015F, YC2016N, YC2016F, YC2017 and YC2018) from 4 year-classes were used in this study. YC2017 and YC2018 are Norwegian populations, and no introgression occurred in those years, hence no letter was appended to them.

The phenotype studied is categorical gill score (0–5) [11] of Atlantic salmon during a natural AGD outbreak, with score 0 indicating no infection; 1 when one white spot is on the gill; 2 if there are two to three small mucus patches; 3 established mucus patch covering 20% of the gill area; 4 established lesions covering up to 50% of the gill area; 5 extensive lesions on most of the gill surface. For populations (YC2015N and YC2015F), which had more than one record of gill score, only the first gill score was used because this was available for all of our study populations. In addition, the first infection with AGD has been reported to be genetically distinct from subsequent reinfections [12]. The numbers of individuals and families used in this study are in Table 1.

**Table 1:** Numbers of individuals and families included in the study

Population	Number of families	Number of individuals
YC2015N	273	2464
YC2015F	116	1049
YC2016N	180	2006
YC2016F	70	640
YC2017	275	2911
YC2018	139	2949

Total number of families = 1053; Total number of individuals = 12,019

### Genotypes

The fish were genotyped with a custom 55k SNP chip developed by Nofima in collaboration with SalmoBreed and Mowi. A quality control procedure was applied on all populations jointly: markers and samples with a call rate < 95%, SNPs with a minor allele frequency < 1%, and SNPs with Hardy Weinberg p value (Fisher’s exact test) < 10^–25^ were discarded. Finally, only samples with a heterozygote frequency > 0.25 and < 0.45 were retained to limit the impact of poor-quality samples [13]. These procedures were done with the Plink software [14]. Sporadically missing genotypes were imputed using Beagle version 5.4 [15] with the following parameters: *window* = *20* (to split each chromosome into 20-Mb segments), *overlap* = *5* (the size of the overlaps between windows is set to 5 Mb), *burnin* = *10,* and *iterations* = *50*, and the remaining parameters were set to default. The imputation step was necessary because missing genotypes are problematic for one of the genetic evaluation software (Wombat [16]) that was used in this study. After quality filtering, 50,456 SNPs remained, which is referred to as *all SNPs* hereafter.

### Genome wide association analysis (GWAA) to select SNPs

The following model was used for evaluation of SNP effects:

$$\mathbf{y}=\mathbf{x}\mathbf{b}+\mathbf{Z}\mathbf{u}+\mathbf{e},$$

where $\mathbf{y}$ is the vector of gill scores, $\mathbf{x}$ is the vector of SNP genotypes (coded 0|*AA*, 1|*AG*, 2|*GG*), $\mathbf{b}$ is the allele substitution effect of each SNP, $\mathbf{Z}$ is an incidence matrix relating the phenotype to the residual polygenic effect $\mathbf{u}$, and $\mathbf{e}$ is the vector of the residual environmental effect. $\mathbf{u}\sim N(\boldsymbol{0},\mathbf{G}{\sigma }_{u}^{2})$, $\mathbf{e}\sim N(\boldsymbol{0},\mathbf{I}{\sigma }_{e}^{2})$, where $\mathbf{I}$ is an identity matrix, ${\sigma }_{{\text{e}}}^{2}$ is the residual variance, $\mathbf{G}$ is the genomic relationship matrix (GRM), ${\sigma }_{{\text{u}}}^{2}$ is the additive genomic variance. The GRM was computed using default settings in the GCTA software [19]. To avoid biases that may arise from including the GP validation set in the SNP discovery process [17, 18], the SNP discovery process reflected the cross-validation (see below) scheme used for GP. The cross-validation process used for GP involved a within-population step of randomly selecting one individual per family for the validation set, while the remaining individuals were the reference set. Thereafter, the size of the reference set was increased by adding parallel population (s) from previous year (s) or YC. This whole process was repeated 50 times. Accordingly, the SNP discovery process involved running a GWAA within a population while excluding the validation set with the model described above using the GCTA software [19] (–mlma option). A separate GWAA was then conducted for each population(s) added to the reference set. Then, SNPs with the same sign of allele substitution effect estimates (based on the GWAA results) across all the populations in the reference set were selected, referred to as *subset of SNPs*, and used for GP. This process was repeated 50 times, as in the cross-validation process. The number of *subset of SNPs* varied across the multi-population prediction scenarios studied (Table 2). Additional file 1: Table S1 shows the average number of SNPs for each multi-population scenario studied with *subset of SNPs.* Populations from YC 2015 were excluded from analyses with *subset of SNPs* due to their impact on prediction accuracy based on *all SNPs* (see details in Discussion).

**Table 2:** Multi-population prediction scenarios evaluated for the subset of SNPs

Reference set	Validation
YC2016N + YC2017	YC2017
YC2016F + YC2017	YC2017
YC2016N + YC2016F + YC2017	YC2017
YC2017 + YC2018	YC2018
YC2016N + YC2017 + YC2018	YC2018
YC2016F + YC2017 + YC2018	YC2018
YC2016N + YC2016F + YC2017 + YC2018	YC2018

### Genomic prediction models

The various genomic prediction models used in this study (genomic BLUP (GBLUP), Bayes B and Bayes R) are fully described in Ajasa et al. [8]. Briefly, the prediction models differed in their prior assumptions about SNP effects; GBLUP assumes that all SNPs have an effect from a normal distribution [20]; Bayes B assumes that a fraction of the SNPs (1-π) have an effect coming from a t-distribution, while the remaining fraction π have no effect [21]; Bayes R assumes that the SNP effects come from a series of normal distributions with a varying degrees of variance [22].

A general presentation of the GP model used in this study is:

$$\mathbf{y}=1\upmu +\mathbf{W}\mathbf{q}+\sum_{i}^{m}{\mathbf{X}}_{i}{\upbeta }_{i}{\uptau }_{i}+\mathbf{e},$$

where $\mathbf{y}$ is the vector of gill scores, $\upmu$ is the overall mean, $\mathbf{W}$ is the incidence matrix relating the phenotype to the fixed effect of YC $\mathbf{q}$, ${\mathbf{X}}_{i}$ is the column vector of marker genotype codes at SNP $i$, ${\upbeta }_{i}$ is the allele substitution effect for SNP $i$, ${\uptau }_{i}$ is a 0/1 indicator variable for SNP $i$*,* $m$ is the number of markers, and $\mathbf{e}$ is the vector of random residual effects. The indicator variable (${\uptau }_{i}$) is 1 for all markers in SNPBLUP (equivalence with GBLUP has been shown by Habier et al. [20] and others), while for Bayes B and Bayes R, it can be 0 or 1.

In addition to these GP models, a multitrait GBLUP (MTGBLUP) model was used, which treats the same trait measured in different populations as a separate but correlated trait, thus allowing for the genetic correlation between populations to differ from 1. The MTGBLUP model fitted was:

$$\left[\begin{array}{c}{\mathbf{y}}_{1}\\ \begin{array}{c}{\mathbf{y}}_{2}\end{array}\end{array}\right]=\left[\begin{array}{cc}1& 0\\ 0& 1\end{array}\right]\left[\begin{array}{c}{\upmu }_{1}\\ \begin{array}{c}{\upmu }_{2}\end{array}\end{array}\right]+\left[\begin{array}{cc}{\mathbf{W}}_{1}& 0\\ 0& {\mathbf{W}}_{2}\end{array}\right]\left[\begin{array}{c}{\mathbf{q}}_{1}\\ \begin{array}{c}{\mathbf{q}}_{2}\end{array}\end{array}\right]+\left[\begin{array}{cc}{\mathbf{K}}_{1}& 0\\ 0& {\mathbf{K}}_{2}\end{array}\right]\left[\begin{array}{c}{\mathbf{u}}_{1}\\ \begin{array}{c}{\mathbf{u}}_{2}\end{array}\end{array}\right]+\left[\begin{array}{c}{\mathbf{e}}_{1}\\ \begin{array}{c}{\mathbf{e}}_{2}\end{array}\end{array}\right],$$

$$\begin{array}{c}{\mathbf{u}}_{{1}}\\ {\mathbf{u}}_{\boldsymbol{2}}\end{array}\sim N\left[\left(\begin{array}{c}0\\ 0\end{array}\right),\left(\begin{array}{cc}{\mathbf{G}}_{\boldsymbol{1}}{\sigma }_{{u}_{{1}}}^{{2}}&{\mathbf{G}}_{{\boldsymbol{1},\boldsymbol{2}}}{\sigma }_{{u}_{{1,2}}}\\ {\mathbf{G}}_{\boldsymbol{2,1}}{\sigma }_{{u}_{\mathrm{2,1}}}& {\mathbf{G}}_{\boldsymbol{2}}{\sigma }_{{u}_{2}}^{2}\end{array}\right)\right]\,\mathrm{with }\,{\mathbf{G}}_{\boldsymbol{1,2}}={\mathbf{G}}_{\boldsymbol{2,1}},$$

$${\mathbf{G}}_{\boldsymbol{2,1}}= \left[\begin{array}{c}\frac{{\mathbf{Z}}_{\boldsymbol{2}}{{\mathbf{Z}}_{\boldsymbol{1}}^{\mathbf{^{\prime}}}}}{\sqrt{\sum_{i}^{m}2{p}_{2i}\left(1-{p}_{2i}\right)}\sqrt{\sum_{i}^{m}2{p}_{1i}\left(1-{p}_{1i}\right)} }\end{array}\right],$$

$$\mathbf{G}=\left[\begin{array}{c}\begin{array}{cc}{\mathbf{G}}_{\boldsymbol{1}}& {\mathbf{G}}_{\boldsymbol{1,2}}\\ {\mathbf{G}}_{\boldsymbol{2,1}}& {\mathbf{G}}_{\boldsymbol{2}}\end{array}\end{array}\right],$$

$$var\left[\begin{array}{c}{\mathbf{e}}_{\boldsymbol{1}}\\ {{\varvec{e}}}_{\boldsymbol{2}}\end{array}\right]=\left[\begin{array}{cc}{\sigma }_{e1}^{2}& 0\\ 0& {\sigma }_{e2}^{2}\end{array}\right],$$

where ${\mathbf{y}}_{\boldsymbol{1}}$ and ${\mathbf{y}}_{\boldsymbol{2}}$ are the vectors of phenotypes for gill score in populations 1 and 2, ${\upmu }_{1}$ and ${\upmu }_{2}$ are the means for gill score in populations 1 and 2, ${\mathbf{W}}_{\boldsymbol{1}}$ and ${\mathbf{W}}_{2}$ are the incidence matrices relating the phenotypes to the fixed effect of YC ${\mathbf{q}}_{\boldsymbol{1}}$ and ${\mathbf{q}}_{\boldsymbol{2}}$ for populations 1 and 2, ${\mathbf{K}}_{\boldsymbol{1}}$ and ${\mathbf{K}}_{\boldsymbol{2}}$ are the incidence matrices relating the phenotypes to the additive genetic effect ${\mathbf{u}}_{\boldsymbol{1}}$ and ${\mathbf{u}}_{\boldsymbol{2}}$ of gill score in populations 1 and 2, ${\sigma }_{e1}^{2}$ and ${\sigma }_{e2}^{2}$ are the residual variances for gill score in populations 1 and 2, respectively. ${\sigma }_{{u}_{1}}^{2}$ and ${\sigma }_{{u}_{2}}^{2}$ are the additive genetic variances for gill score in populations 1 and 2, and ${\sigma }_{{u}_{\mathrm{1,2}}}$ is the additive genetic covariance for gill score between populations 1 and 2. ${\mathbf{G}}_{\boldsymbol{1}}$ and ${\mathbf{G}}_{\boldsymbol{2}}$ are the genomic relationship matrices of populations 1 and 2, respectively, constructed by using Method 1 of VanRaden [23]. ${\mathbf{Z}}_{\boldsymbol{1}}$ and ${\mathbf{Z}}_{\boldsymbol{2}}$ are centred genotype matrices for individuals in populations 1 and 2, ${p}_{1}$ and ${p}_{2}$ are the allele frequencies at SNP $i$ in populations 1 and 2, respectively. $\mathbf{G}$ is the genomic relationship matrix of all individuals in both populations, constructed following Zhou et al. [24] and Wientjes et al. [25]. This matrix and its inverse were computed with R [26] and then included as a user-defined genomic inverse relationship matrix in Wombat [16]. This model also allows for estimation of the genetic correlation between populations, which was estimated here in a pairwise manner due to computational and convergence issues. The GBLUP, Bayes B, and Bayes R models were implemented with Wombat [16], BGLR [27], and GCTB [28], respectively, as in Ajasa et al. [8].

### Multi- and across-population accuracy and bias of predictions

As stated earlier, multi-population accuracy of GP was evaluated by cross-validation, mimicking sib testing, as described in Ajasa et al. [8] for within-population cross-validation, except that the size of the reference set was increased by including parallel population (s), i.e. populations from previous years or YC. The accuracy was calculated as given by the equation below.

The average values of the variance component estimates of the populations in the reference set were used for the prediction of genomic estimated breeding values (GEBV) (with the GBLUP model) in Wombat [16]. When all populations were included in the reference set, the genetic (co)variance matrix of the MTGBLUP model was bent [29] in order to make it positive definite. To speed up the computational process of the Bayesian models, each loop of the cross-validation run was computed on different nodes (parallel computing) of the Norwegian University of Life Sciences (NMBU) Orion computing cluster.

Accuracy was computed as the correlation of the phenotype and GEBV, divided by the square root of the pedigree-based estimate of heritability:

$$accuracy \left(r\right)=\frac{cor\left(gill score,\left(g\right)ebv\right)}{\sqrt{{h}_{ped}^{2}}}.$$

Prediction bias (scale) of GEBV was derived from the regression coefficient of the phenotype on the GEBV [30], with values lower than 1 indicating inflation (overdispersion) of GEBV and values higher than 1 indicating deflation (underdispersion) of GEBV:

$$Prediction \,bias =\frac{cov (gill score, \left(g\right)ebv)}{var\left((g\right)ebv)},$$

where ${h}_{ped}^{2}$ is the pedigree-based heritability estimate, which were 0.24, 0.17, 0.21, and 0.19 for YC2016N, YC2016F, YC2017, and YC2018, respectively [8]. The cross-validation process was repeated 50 times to estimate the mean accuracy and bias, and the standard deviation between the 50 replicates was taken as the standard error (SE). Accuracy and bias of the various GP models in this study were evaluated with *all SNPs* and with the *subset of SNPs*.

For across-population GP, parallel population(s) from previous year(s) or YC were used to predict the current population, which did not require cross-validation. The MTGBLUP model was not used here because it requires prior knowledge of the genetic covariance between populations to yield unbiased estimates, and this is not known in most practical situations. Accuracy and bias of predictions were estimated in the same manner as described above but only one replicate was available for each validation population.

### Genetic distance and persistence of LD phase between populations

The pairwise F_st_ between populations was estimated using *all SNPs* based on Weir and Cockerham’s method [31] in Plink [14]. To estimate the persistency of LD phase between adjacent markers across populations, the *r* statistics (as in de Roos et al. [4]) between pairs of markers was estimated with Plink [14] using the parameters –r –ld-window 2 –ld-window-r2 0 –ld-window-kb 1000. The parameter –ld-window 2 indicates that only two SNPs should be considered in each window, –ld-window-kb 1000 sets the maximum distance between SNPs in a window used for the estimation of *r* to 1000, –ld-window-r2 0 was specified because Plink estimates *r*^2^ first before estimating *r* and by default Plink only considers *r*^2^ values > 0.2. Correlations across populations of estimated *r* values between adjacent SNPs using *all SNPs*, were estimated with R [26].

## Results

### Genetic correlations between populations

The estimates of the genetic correlations for AGD resistance between populations ranged from 0.19 to 0.99 (Table 3), with the highest estimate (0.99) being between YC2016F and YC2017 and the lowest (0.19) being between YC2015N and YC2016F. However, most of these estimates have large standard errors, with a few estimates being significantly different from zero. For YC2015N and YC2015F, the software could not estimate standard errors, probably due to the small sample size and low heritability [8].

**Table 3:** Estimates of genetic correlation for AGD resistance between populations ± standard error

Population	YC2015F	YC2016N	YC2016F	YC2017	YC2018
YC2015N	0.86^†^	0.33 ± 0.35	0.19 ± 0.69	0.45 ± 0.38	0.21 ± 0.34
YC2015F		0.64 ± 0.65	0.33 ± 0.71	0.62 ± 0.59	0.97 ± 0.62
YC2016N			0.91 ± 0.51	0.99 ± 0.23*	0.65 ± 0.26*
YC2016F				0.99 ± 0.48*	0.98 ± 0.57
YC2017					0.95 ± 0.16*

^†^Software failed to estimate standard errors

*Significantly different from zero

### Within-population prediction accuracy and genetic parameters

Table 4 shows estimates of genetic parameters and within-population accuracy for AGD resistance. Heritability estimates were around 0.2 for each population, while the within-population accuracy estimates of breeding values ranged from 0.47 to 0.76.

**Table 4:** Estimates of genetic parameters and of within-population accuracy and bias of breeding value predictions for AGD resistance using pedigree and *all SNPs*

Population	*All SNPs*					Pedigree		
	${{\varvec{\sigma}}}_{{\varvec{u}}}^{2}$** ± SE**	${{\varvec{\sigma}}}_{{\varvec{e}}}^{2}$** ± SE**	**h**^**2**^** ± SE**	**acc. ± SE**	**bias ± SE**	**acc. ± SE**		**bias ± SE**
YC2016N	0.43 ± 0.07	1.42 ± 0.06	0.23 ± 0.03	0.56 ± 0.12	0.91 ± 0.21	0.47 ± 0.12		0.95 ± 0.28
YC2016F	0.33 ± 0.11	1.36 ± 0.11	0.19 ± 0.06	0.54 ± 0.38	1.05 ± 0.78	0.47 ± 0.38		1.13 ± 0.94
YC2017	0.36 ± 0.05	1.34 ± 0.43	0.21 ± 0.03	0.73 ± 0.12	1.01 ± 0.19	0.59 ± 0.12		1.01 ± 0.24
YC2018	0.30 ± 0.04	1.12 ± 0.03	0.21 ± 0.03	0.76 ± 0.14	1.02 ± 0.21	0.56 ± 0.15		0.99 ± 0.28

Results as reported in Ajasa et al. [8]

${\sigma }_{u}^{2}$: additive genomic variance, ${\sigma }_{e}^{2}$: residual variance, SE: standard error, h^2^: heritability, acc.: accuracy

### Multi-population accuracy and bias of predictions

**Fig. 1:**
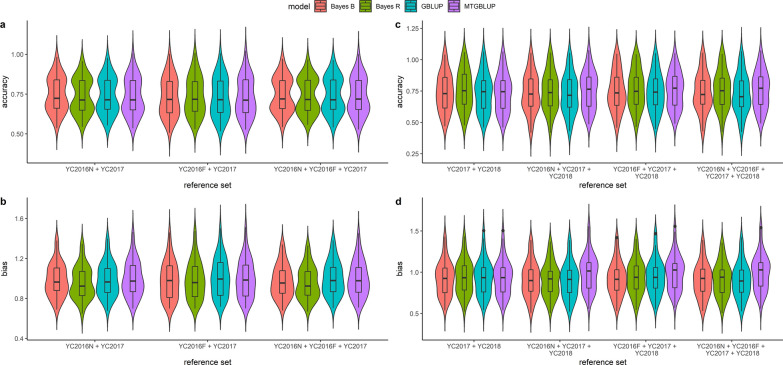
Multi-population accuracy and bias of predictions using *all SNPs* and different genomic prediction models for validation populations YC2017 (**a**, **b**) and YC2018 (**c**, **d**)

Multi-population prediction accuracies with *all SNPs* are shown in Fig. 1 and Additional file 1: Table S2. The prediction accuracy ranged from 0.47 to 0.76 and the bias ranged from 0.90 to 1.48. There was no significant increase in prediction accuracy by combining multiple populations in the reference set, compared to the regular within-population prediction (Table 4). In fact, in some cases, we saw a reduction in prediction accuracy when combining populations, for example when combining YC2015N, YC2015F, YC2016N, YC2016F, and YC2017 in one reference set (see Additional file 1: Table S2). Combining multiple populations in the reference set had no impact on standard errors, as otherwise would be expected from an increased reference set size, and in almost all cases the GP models had a similar performance. In general, the prediction bias of GEBV was close to 1 when combining populations, indicating that combining multiple populations did not affect the scale of the GEBV.

**Fig. 2:**
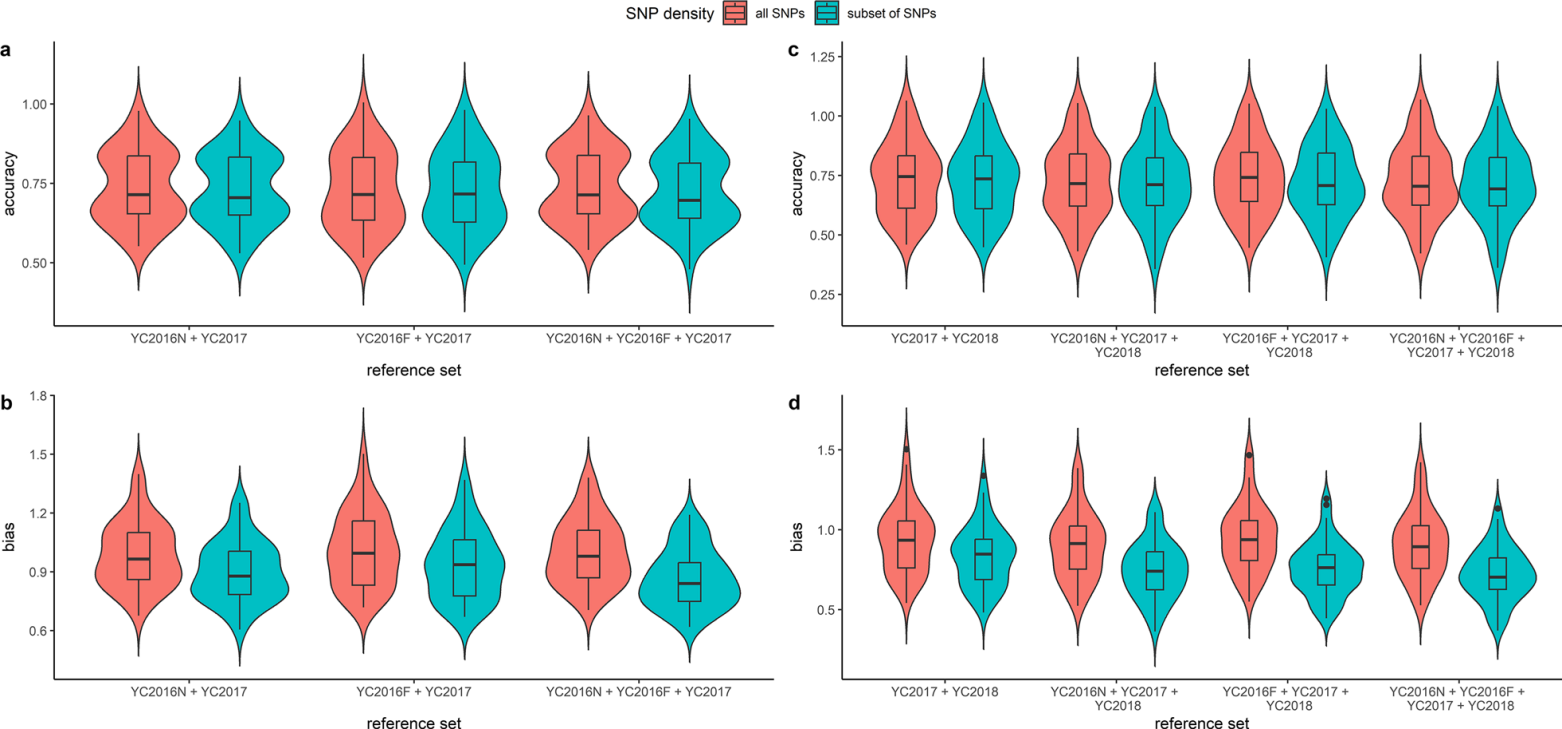
Multi-population accuracy and bias of predictions using *all SNPs* and *subset of SNPs* for validation populations YC2017 (**a**, **b**) and YC2018 (**c**, **d**)

Figure 2 and Additional file 1: Table S3 show prediction accuracies based on *subset of SNPs*. The prediction accuracy ranged from 0.72 to 0.73 and the prediction bias ranged from 0.72 to 0.94. Compared to using *all SNPs,* using *subset of SNPs* did not increase the prediction accuracy. In addition, the predictions were generally highly biased.

### Across-population accuracy and bias of predictions

**Fig. 3:**
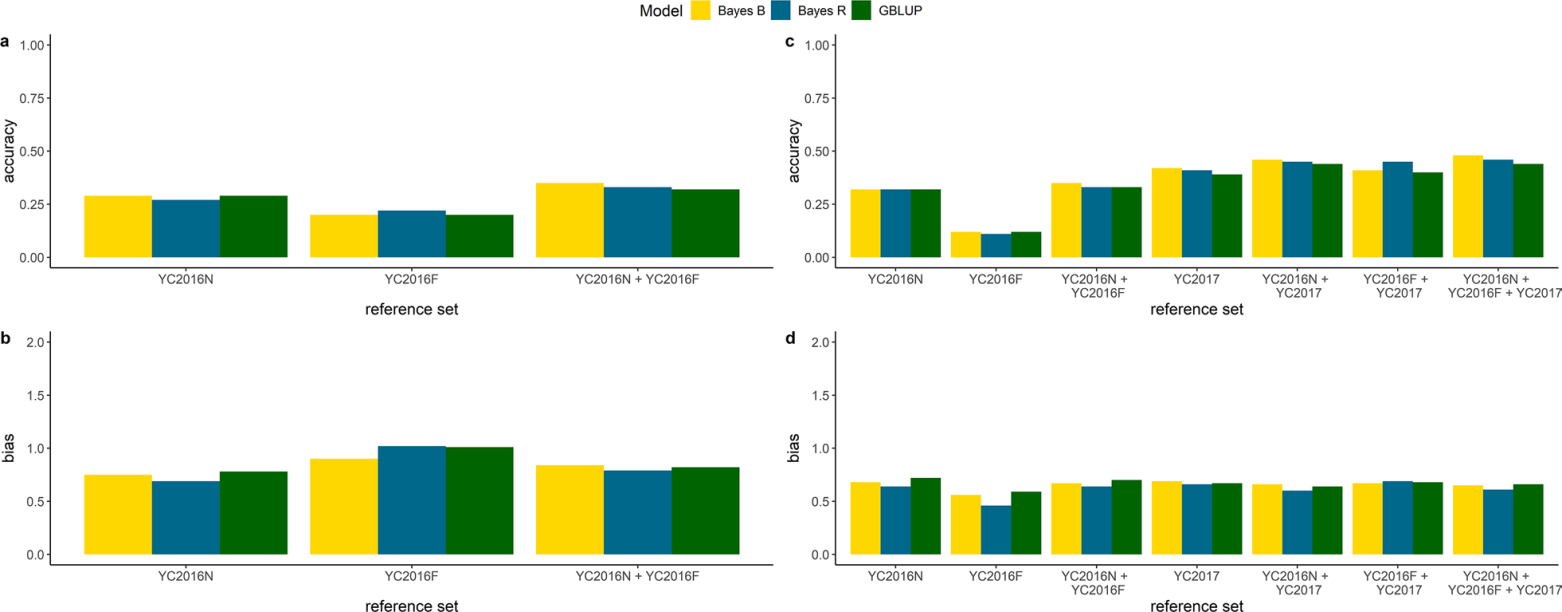
Across population accuracy and bias of predictions using *all SNPs* and different genomic prediction models for validation populations YC2017 (**a**, **b**) and YC2018 (**c**, **d**)

Figure 3 and Additional file 1: Table S4 show the across-population prediction accuracies. For the across-populations scenarios evaluated, prediction accuracies ranged from − 0.04 to 0.49, which was significantly lower than the accuracy of multi-population predictions. In general, across-population prediction accuracies were greater for populations that had high genetic correlation estimates. For example, the genetic correlation between YC2017 and YC2018 was estimated at 0.95 and the across-population predictions for YC2018 using YC2017 as the reference set was 0.39. The performance of the nonlinear models did not differ significantly from that of GBLUP. In most cases, GEBV were inflated.

### Genetic distances between populations

The mean genetic distances between populations are in Table 5. The estimates ranged from 0.03 to 0.07, with YC2017 and YC2018 being the most genetically similar, while YC2015N and YC2015F, and YC2015F, and YC2018 were the least related. In general, there was no strong genetic differentiation between the populations. Additional file 1: Table S5 shows the correlation of minor allele frequencies between populations.

**Table 5:** Mean estimates of F_st_ between populations

Population	YC2015F	YC2016N	YC2016F	YC2017	YC2018
YC2015N	0.07	0.04	0.05	0.05	0.04
YC2015F		0.06	0.04	0.06	0.07
YC2016N			0.05	0.04	0.05
YC2016F				0.05	0.06
YC2017					0.03

### Persistency of LD phase across populations

**Fig. 4:**
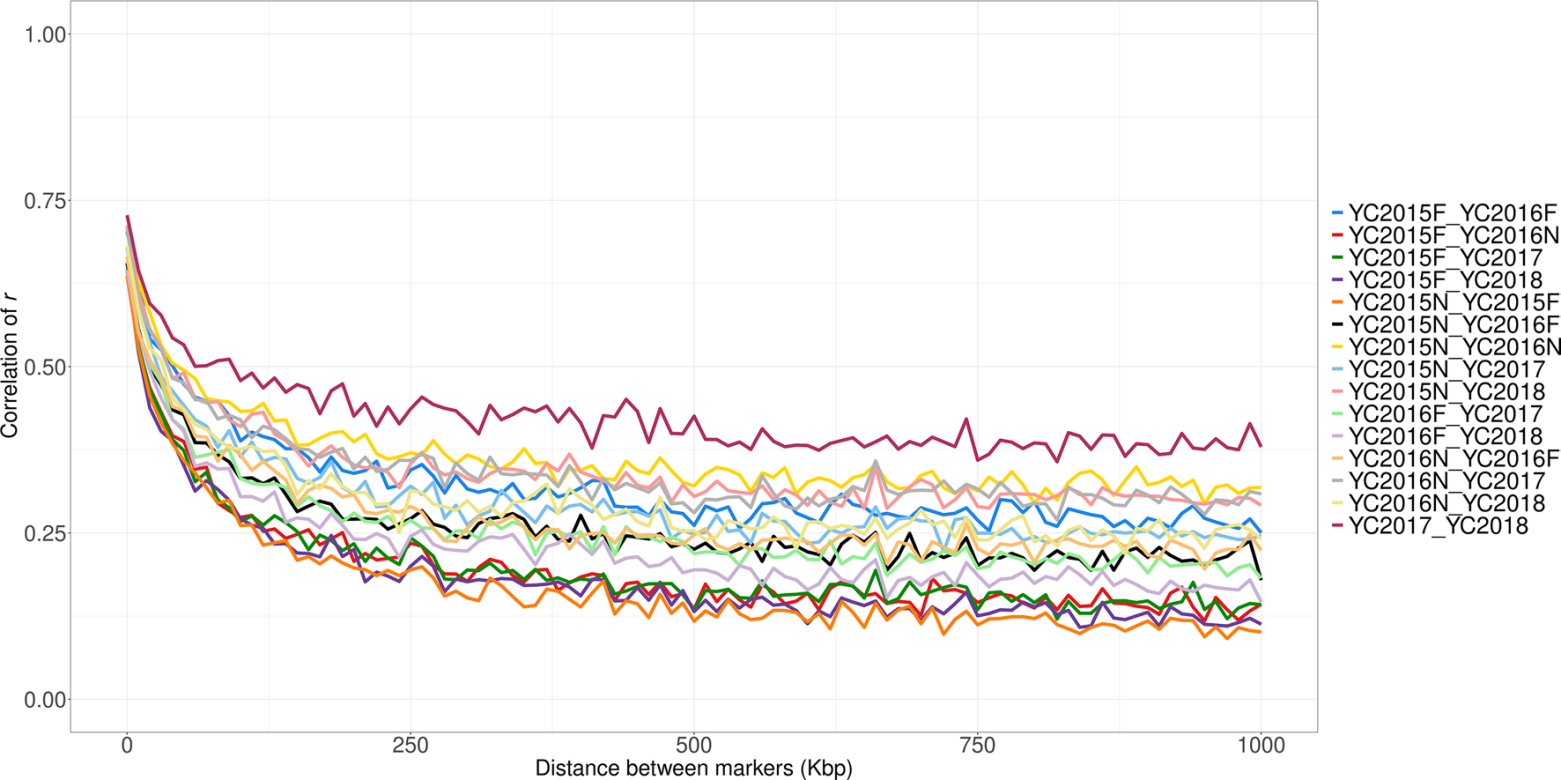
Persistency of LD phase between populations measured by the correlation of *r* between populations

The average distance between adjacent SNPs was 43 kb. The estimates of persistency of LD phase across populations, measured as the correlation of *r* between pairs of adjacent SNPs (across populations), are in Table 6. In principle, the correlation of *r* can range from -1 to 1, with a high value indicating that the two populations share the same haplotypes and a low value indicating that the haplotypes are different. Correlation of *r* estimates were in general similar in size and low. A decline in these estimates with genetic distance is illustrated in Fig. 4. For all pairs of populations studied, the correlation of *r* decreased steadily with distance up to an inter-marker distance of about 750 kb (Fig. 4), at which point it remained fairly constant. The highest correlation of *r* was observed between YC2017 and YC2018 and this is consistent with the recent mixing history of these populations.

**Table 6:** Persistency of LD phase measured by the correlation of *r* between adjacent SNPs across populations

Population	YC2015F	YC2016N	YC2016F	YC2017	YC2018
YC2015N	0.51	0.62	0.56	0.57	0.61
YC2015F		0.52	0.61	0.53	0.52
YC2016N			0.57	0.60	0.58
YC2016F				0.56	0.54
YC2017					0.65

## Discussion

The main aim of this study was to evaluate the impact of combining Atlantic salmon populations on the accuracy of multi- and across-population predictions of breeding values for AGD resistance. Different GP models and a SNP-selection approach were evaluated.

### Genetic correlations between populations

Genetic correlations between populations are usually estimated with a multi-trait model that treats the same trait measured in different populations as different but correlated traits. In this study, AGD resistance was recorded for different populations in different years, thus a high or low genetic correlation between populations could be due to genetic or environmental reasons. The genetic cause could arise from the same or different causative variants segregating in different populations, the same or different causative effect sizes, and the same or different pattern of LD between markers and causal loci in different populations [25]. The environmental cause could be due to the same or different intensity of AGD outbreaks in different years, the same or different water temperatures, the same or different salinity, the same or different water quality, the same or differential accuracy of recording across years and so on. The fixed effect of YC in the model is expected to account for these variations in environmental factors, but if genotype-by-environment interactions (GxE) with any of these factors are present, they will reduce the genetic correlation between populations. Most of the genetic correlation estimates in our study had large standard errors, which limits the ability to make reasonable inference from the estimates. Nevertheless, the high genetic correlation that we observed between YC2016N and YC2017, and between YC2017 and YC2018 indicates that the same trait is measured in these years i.e., little environmental variation or no GxE, and the same causal loci are probably segregating in these populations.

### Multi-population prediction

Several studies, e.g. [32, 33] have highlighted the importance of a large reference population for genomic prediction. In aquaculture, many factors can complicate the creation of a sufficiently large reference population, such as the expensive nature of disease challenge trials, and the loss of fish during phenotype recording. Another important factor is the design or structure of aquaculture breeding programs, in which the number of fish per family may be limited due to cost considerations. A good strategy to increase the size of the reference population could be to combine data from multiple populations that may be separated by space or time.

The similarity of SNP effects between populations is usually reflected in the genetic correlation estimate [34, 35]. However, since SNP effects may not be correlated between populations, a multi-trait GBLUP model was used, which allows the genetic correlation between populations to differ from 1. In all of the models we investigated, only the MTGBLUP model had consistent performance, in terms of not being worse than the within-population prediction accuracy in all multi-population scenarios examined. This result is consistent with those of Carillier et al. [36], who observed no difference in accuracy when performing within-population or multiple-population prediction of breeding values with a multi-trait model. Several authors [33, 37, 38] have suggested that when two or more populations have a high genetic correlation, combining them in one reference set can potentially result in an increase in prediction accuracy, perhaps because intuitively the genetic correlation should give an indication of persistency of LD phase between markers and QTL across populations. Surprisingly, in spite of the high genetic correlation estimate between some populations, for example between YC2017 and YC2018 (0.95), combining them in one reference set did not result in an increase in prediction accuracy. An explanation for this could be that a high genetic correlation between populations does not guarantee that the LD phase between markers and causal variants are the same across populations, as seen for instance by the low persistency of LD phase across populations (Table 6) in this study. In a simulation study, Wientjes et al. [39], also noted that the estimates of genetic correlation between populations is not affected by the persistency of LD phase across populations. Hence, the small or negligible impact of increasing the reference set with other populations on prediction accuracy in our study could be the result of the low persistency of LD phase across populations. Consistent with this, some of the studies [40, 41] that have reported a substantial increase in prediction accuracy when combining multiple populations have observed a high persistency of LD phase (0.86–0.97) across the populations studied. Thus, knowledge of LD phase across populations rather than of the genetic correlation may be important for assessing the value of multi-population prediction.

Another explanation for the inconsequential impact of multi-population prediction on accuracy of prediction could be the cross-validation scheme that was used in this study (see Methods). Based on selection index theory, when information from close and distant relatives are available, more weight is usually given to the former [42]. Therefore, when we combined multiple populations, more weight was likely given to the information coming from sib relationships between the validation and reference set (at least for the MTGBLUP model) than to information from distant relationship (i.e. from other populations) and this was probably exacerbated by the low persistency of LD phase across the studied populations. For the majority of cases (see Additional file 1: Table S2), adding populations from the year 2015 to the reference set, particularly YC2015N (probably due to its comparatively large sample size), reduced the accuracy of prediction (with the exception of the MTGBLUP model) compared to the within-population prediction. This can be due to the seasonal variation in the outbreak of AGD, with the first infection of AGD for the year 2015 recorded in September, compared to November in the other years (2016, 2017, 2018). This is probably reflected in the heritability estimate from year 2015 (~ 0.1) vs others years (~ 0.2). Hence the trait measured in 2015 might differ from that recorded in other years. This illustrates one of the practical constraints that may restrict the combining of multiple populations in aquaculture breeding programs.

Some authors [5, 43] have argued for the use of nonlinear models in a multi-population setting because they can potentially better emphasize markers that are very close to the causal variants, thereby resulting in more consistent LD phase of markers and causal variants across populations. Indeed, a number of studies, e.g. [38, 44], have reported better performance from using Bayesian models in such instances. However, in our study, the nonlinear models that we examined did not perform any better than their linear counterparts. A probable reason for the discordance of our results with others is that the traits examined in the referred studies [38, 44] are in part controlled by QTL with large effects, whereas the trait that we examined here is polygenic [45], i.e. controlled by a large number of genes, each with a small effect. Similar to our study, Calus et al. [46] reported no benefit of nonlinear models for a largely polygenic trait.

SNPs with effects of the same sign/direction across populations are usually considered to be true effects [47], and some studies [6, 7] have shown promising results from using SNPs that have the same sign or direction across populations for multi- or across-population GP. However, in our study, using *subset of SNPs* had a limited or reduced impact on multi-population accuracy compared with using *all SNPs*, and in general, the resulting predictions were highly biased. This bias could be because *subset of SNPs* were identified in the same population as the reference set [17, 18]. Excluding the prediction validation set in the SNP discovery process was done to circumvent this problem, however, our results indicate that this was not successful. Similarly, Fraslin et al. [48] reported highly biased predictions when using top SNPs that were identified using a leave one group out cross-validation. Hence, a more appropriate design might be to have a separate population for GWAA and GP, but this is not relevant for the current Atlantic salmon breeding program where population size is limited due to cost considerations. Nevertheless, Ros-Freixedes et al. [49] did not observe biased predictions when using variants identified from the same population as the reference set for GP. The discordance between our results using the *subset of SNPs* and those of van den Berg et al. [7], where an increase in prediction accuracy was achieved using subsets of SNPs for multi-population prediction, could be due to the relatively smaller population sizes and smaller SNP density in our study; a SNP may be of the same sign across populations due to chance [47] and not be a true effect, and with a larger size of each population and sequence information, GWAA may identify causal variants or SNPs that are very close to the causal variant, and consequently of the same sign across populations. However, the requirement for a larger sample size for within-population GWAA partly defeats the purpose of multi-population prediction.

### Across-population prediction

Across-population predictions would be invaluable for both field and disease challenge trials in aquaculture breeding programs, as they would reduce the need for phenotyping. This is particularly important when selecting for resistance to diseases such as AGD in Norway, where AGD outbreaks are still rather rare and unpredictable.

A major problem in across-population prediction arises when the causal variants are unknown, forcing the need to rely on LD between markers and QTL. As mentioned before, and also relevant here, the phase between markers and QTL might not be the same across populations. And even if they are the same, imperfect LD between markers and QTL might still be a problem. There may also be different causative loci segregating in different populations [1], but this is less likely in our dataset due to the genetic similarity of our study populations (Table 5). Within population, the problem of imperfect LD between markers and QTL is compensated for by the co-segregation of markers and QTL due to recent familial relationships [43]. In less related populations, such as in across-population prediction, this familial relationship does not exist, leading to the need to focus on models that can capture LD between markers and QTL better. As stated earlier, several authors [5, 20, 43] have proposed the use of nonlinear models for such cases, as they can place more emphasis on markers that are in LD with the QTL (and less on relationships) across populations, which thus should result in better prediction accuracy. However, the results reported here do not support this and, in all the scenarios we examined, there were no significant differences between the models. Similar observations were also made by Calus et al. [46] in layer lines. In addition, our results agree with those of a simulation study by van den Berg et al. [35], who demonstrated that nonlinear models perform similarly as GBLUP in across-population prediction when the number of QTL affecting a trait is large. This also supports our conclusion in a previous study (Ajasa et al. [8]), that AGD resistance is likely polygenic.

The across-population prediction accuracies that we observed with *all SNPs* were somewhat proportional to the genetic correlation between the populations. This is in line with Wientjes et al. [25], who noted that knowledge of the genetic correlation between populations is important when predicting across populations, as this can give an indication of the upper limit of across-population prediction accuracy. Hence, it appears that genetic correlation estimates between populations may have different impacts in a multi- and across-population setting. Further research is thus needed to understand what the genetic correlation between populations really measures in this regard and how it affects multi- and across-population prediction.

The prediction accuracy obtained for predicting across populations or from training on distant relatives was described by Clark et al. [42] as ‘baseline accuracy’, and they showed that, in some cases, this accuracy can be similar to or even higher than the accuracy of pedigree-based predictions on related individuals. In our study however, the accuracy obtained for predicting across populations using *all SNPs* was substantially smaller than the pedigree-based within-population prediction accuracy (Table 4), even with a large reference set. Hence, across-population predictions have limited use for Atlantic salmon breeding purposes.

### Persistency of LD phase

The first step before combining multiple populations should be to assess the persistency of LD phase between markers and QTL across populations, as this may give an indication of the potential impact on prediction accuracy of combining multiple populations [50]. Since QTL are usually not known, this information can only be inferred from markers [50].

Numerous studies have examined the persistency of LD phase across different cattle [4, 51], sheep [52], goat [53], chicken [50, 54] and pig [55, 56] populations but, to our knowledge, this is the first study for an aquaculture species. The persistency of LD phase between adjacent markers across populations (Table 6) ranged from 0.51 to 0.65, while the genetic distance (Table 5) ranged from 0.03 to 0.07. Taken together, these results suggest that there is an inverse relationship between the persistency of LD phase and genetic distance between populations, which is consistent with previous observations by de Roos et al. [4] and Andreescu et al. [50], who concluded that persistency of LD phase across populations gives an important indication of the genetic relationship between populations. However, given the high genetic similarity of the populations in our study (Table 5), the low persistency of LD phase observed between adjacent markers was quite surprising and lower than those observed in cattle [4], pig [57], sheep [52] and chicken [54] (0.78–0.97) at a similar distance. A possible explanation for this is the typical structure of aquaculture breeding programs, which usually have relatively many full-sib families tested in each generation to avoid inbreeding, resulting in the presence of many haplotypes in a population. In addition, Atlantic salmon was domesticated only recently. An alternative explanation could be the admixture history of these populations, which may have resulted in the introduction of new haplotypes into each population. This, coupled with genetic drift [8], might have amounted to an increase in haplotype variance across populations.

## Conclusions

We found low persistency of LD phase across populations, despite the close genetic relationship between our study populations. This indicates that combining populations may not increase in prediction accuracy or power of GWAA. Indeed, we found no increase in prediction accuracy when these populations were combined for GP across the various models studied. Our study indicates that a low persistency of LD phase is a major problem that hinders multi- or across-population prediction and that a close relationship and/or high genetic correlation do not guarantee an increase in accuracy from multi-population prediction.

### Supplementary Information

**Additional file 1: Table S1.** Average number of SNPs for each multi-population scenario studied using *subset of SNPs*. **Table S2.** Multi-population accuracy and bias of predictions using *all SNPs*. **Table S3.** Multi-population accuracy and bias of predictions using *subset of SNPs*. **Table S4.** Across-population accuracy and bias of predictions using *all SNPs*. **Table S5.** Correlation of minor allele frequencies between populations.

## Data Availability

The data used in this study were provided by Mowi Genetics AS and are not publicly accessible.
