# Supplementary material for: Accuracy of genomic prediction using multiple Atlantic salmon populations
Source: Genet Sel Evol. 2024 May 15;56:38. doi: 10.1186/s12711-024-00907-5 (PMC11094890; doi:10.1186/s12711-024-00907-5)
Supplement: Supplementary file 1 — Additional file 1: Table S1. Average number of SNPs for each multi-population scenario studied using subset of SNPs. Table S2. Multi-population accuracy and bias of predictions using all SNPs. Table S3. Multi-population accuracy and bias of predictions using subset of SNPs. Table S4. Across-population accuracy and bias of predictions using all SNPs. Table S5. Correlation of minor allele frequencies between populations. [file 12711_2024_907_MOESM1_ESM.docx]

Table S1: Average number of SNPs for each multi-population scenario studied using subset of SNPs

| Informant | Average number of SNPs with same sign |
| --- | --- |
| YC2016N+YC2017 | 26395.3 |
| YC2016F+YC2017 | 25893.9 |
| YC2016N+ YC2016F+ YC2017 | 13953 |
| YC2017+ YC2018 | 26747.5 |
| YC2016N+YC2017+ YC2018 | 14449.5 |
| YC2016F+YC2017+ YC2018 | 13977.2 |
| YC2016N+ YC2016F+ YC2017+ YC2018 | 7742.28 |

Table S2: Multi-population accuracy and bias of predictions using all SNPs

| Informants | Validation | GBLUP | | MTGBLUP | | Bayes B | | Bayes R | |
| --- | --- | --- | --- | --- | --- | --- | --- | --- | --- |
|  |  | accuracy ± SE | bias ± SE | accuracy ± SE | bias ± SE | accuracy ± SE | bias ± SE | accuracy ± SE | bias ± SE |
| YC2015N + YC2015F + YC2016N + YC2016F | YC2016N | 0.52 ± 0.14 | 1.03 ± 0.29 | 0.56 ± 0.13 | 0.90 ± 0.22 | 0.52 ± 0.14 | 1.11 ± 0.32 | 0.51 ± 0.13 | 1.09 ± 0.31 |
| YC2015N + YC2015F + YC2016N | YC2016N | 0.52 ± 0.13 | 1.05 ± 0.29 | 0.55 ± 0.13 | 0.90 ± 0.22 | 0.51 ± 0.13 | 1.13 ± 0.33 | 0.51 ± 0.13 | 1.11 ± 0.31 |
| YC2015N + YC2016N | YC2016N | 0.51 ± 0.13 | 1.11 ± 0.30 | 0.55 ± 0.13 | 0.90 ± 0.22 | 0.51 ± 0.13 | 1.11 ± 0.32 | 0.51 ± 0.13 | 1.08 ± 0.30 |
| YC2015F + YC2016N | YC2016N | 0.55 ± 0.13 | 0.98 ± 0.24 | 0.56 ± 0.12 | 0.90 ± 0.21 | 0.55 ± 0.13 | 0.98 ± 0.25 | 0.56 ± 0.12 | 0.98 ± 0.24 |
| YC2015N + YC2015F + YC2016N + YC2016F | YC2016F | 0.58 ± 0.39 | 1.16 ± 0.85 | 0.57 ± 0.39 | 1.08 ± 0.77 | 0.58 ± 0.39 | 1.29 ± 0.94 | 0.57 ± 0.39 | 1.24 ± 0.90 |
| YC2015N + YC2015F + YC2016F | YC2016F | 0.52 ± 0.41 | 1.23 ± 1.01 | 0.54 ± 0.38 | 1.05 ± 0.78 | 0.52 ± 0.41 | 1.48 ± 1.21 | 0.51 ± 0.39 | 1.37 ± 1.12 |
| YC2015F + YC2016F | YC2016F | 0.57 ± 0.39 | 1.26 ± 0.92 | 0.55 ± 0.38 | 1.06 ± 0.78 | 0.57 ± 0.39 | 1.29 ± 0.95 | 0.56 ± 0.39 | 1.19 ± 0.88 |
| YC2015N + YC2016F | YC2016F | 0.48 ± 0.39 | 1.44 ± 1.25 | 0.54 ± 0.38 | 1.04 ± 0.77 | 0.48 ± 0.39 | 1.47 ± 1.29 | 0.47 ± 0.39 | 1.26 ± 1.11 |
| YC2016N + YC2017 | YC2017 | 0.74 ± 0.11 | 0.99 ± 0.17 | 0.74 ± 0.12 | 0.99 ± 0.18 | 0.75 ± 0.11 | 0.98 ± 0.16 | 0.74 ± 0.11 | 0.96 ± 0.16 |
| YC2016F + YC2017 | YC2017 | 0.73 ± 0.12 | 1.01 ± 0.19 | 0.73 ± 0.12 | 1.00 ± 0.19 | 0.73 ± 0.12 | 0.98 ± 0.19 | 0.74 ± 0.12 | 0.99 ± 0.19 |
| YC2016N + YC2016F + YC2017 | YC2017 | 0.74 ± 0.11 | 1.00 ± 0.17 | 0.74 ± 0.12 | 0.99 ± 0.18 | 0.75 ± 0.11 | 0.97 ± 0.16 | 0.73 ± 0.12 | 0.95 ± 0.16 |
| YC2015N + YC2015F + YC2016N + YC2016F + YC2017 | YC2017 | 0.63 ± 0.14 | 1.07 ± 0.25 | 0.74 ± 0.12 | 0.99 ± 0.18 | 0.74 ± 0.12 | 1.13 ± 0.19 | 0.74 ± 0.11 | 1.09 ± 0.19 |
| YC2017 + YC2018 | YC2018 | 0.74 ± 0.15 | 0.94 ± 0.21 | 0.76 ± 0.15 | 0.99 ± 0.21 | 0.74 ± 0.15 | 0.93 ± 0.20 | 0.76 ± 0.15 | 0.94 ± 0.21 |
| YC2016N + YC2016F YC2017 + YC2018 | YC2018 | 0.73 ± 0.15 | 0.91 ± 0.21 | 0.76 ± 0.15 | 0.99 ± 0.22 | 0.74 ± 0.16 | 0.91 ± 0.21 | 0.76 ± 0.15 | 0.92 ± 0.20 |
| YC2016N + YC2017 + YC2018 | YC2018 | 0.73 ± 0.16 | 0.90 ± 0.21 | 0.76 ± 0.15 | 0.99 ± 0.22 | 0.73 ± 0.15 | 0.91 ± 0.21 | 0.75 ± 0.15 | 0.91 ± 0.20 |
| YC2016F + YC2017 + YC2018 | YC2018 | 0.74 ± 0.15 | 0.95 ± 0.21 | 0.76 ± 0.15 | 0.99 ± 0.21 | 0.75 ± 0.16 | 0.92 ± 0.21 | 0.76 ± 0.15 | 0.94 ± 0.20 |
| YC2015N + YC2015F + YC2016 + YC2017 + YC2018 | YC2018 | 0.72 ± 0.16 | 0.98 ± 0.24 | 0.76 ± 0.15* | 0.96 ± 0.21 | 0.73 ± 0.16 | 1.01 ± 0.24 | 0.75 ± 0.16 | 1.01 ± 0.24 |

* bent

Table S3: Multi-population accuracy and bias of predictions using subset of SNPs

| Informants | Validation | GBLUP | | Bayes B | | Bayes R | |
| --- | --- | --- | --- | --- | --- | --- | --- |
|  |  | accuracy ± SE | bias ± SE | accuracy ± SE | bias ± SE | accuracy ± SE | bias ± SE |
| YC2016N + YC2017 | YC2017 | 0.73 ± 0.11 | 0.90 ± 0.15 | 0.72 ± 0.11 | 0.79 ± 0.13 | 0.72 ± 0.11 | 0.77 ± 0.13 |
| YC2016F + YC2017 | YC2017 | 0.73 ± 0.12 | 0.94 ± 0.18 | 0.72 ± 0.12 | 0.84 ± 0.16 | 0.72 ± 0.12 | 0.83 ± 0.15 |
| YC2016N + YC2016F + YC2017 | YC2017 | 0.72 ± 0.11 | 0.87 ± 0.15 | 0.71 ± 0.11 | 0.76 ± 0.13 | 0.71 ± 0.11 | 0.75 ± 0.12 |
| YC2017 + YC2018 | YC2018 | 0.73 ± 0.15 | 0.84 ± 0.19 | 0.73 ± 0.15 | 0.76 ± 0.17 | 0.73 ± 0.15 | 0.75 ± 0.17 |
| YC2016N + YC2017 + YC2018 | YC2018 | 0.72 ± 0.16 | 0.75 ± 0.18 | 0.71 ± 0.15 | 0.71 ± 0.17 | 0.72 ± 0.15 | 0.69 ± 0.16 |
| YC2016F + YC2017 + YC2018 | YC2018 | 0.72 ± 0.15 | 0.76 ± 0.17 | 0.72 ± 0.15 | 0.74 ± 0.16 | 0.73 ± 0.15 | 0.73 ± 0.16 |
| YC2016N + YC2016F + YC2017 + YC2018 | YC2018 | 0.72 ± 0.15 | 0.72 ± 0.17 | 0.72 ± 0.15 | 0.71 ± 0.17 | 0.72 ± 0.15 | 0.70 ± 0.16 |

Table S4: Across-population accuracy and bias of predictions using all SNPs

| Informants | Validation | GBLUP | | Bayes B | | Bayes R | |
| --- | --- | --- | --- | --- | --- | --- | --- |
|  |  | accuracy | bias | accuracy | bias | accuracy | bias |
| YC2015N | YC2016N | 0.10 | 0.52 | 0.11 | 0.78 | 0.10 | 0.72 |
| YC2015F | YC2016N | 0.05 | 0.44 | 0.05 | 0.45 | 0.04 | 0.27 |
| YC2015N + YC2015F | YC2016N | 0.11 | 0.60 | 0.11 | 0.60 | 0.11 | 0.59 |
| YC2015N | YC2016F | -0.01 | -0.06 | -0.04 | -0.26 | 0.01 | 0.03 |
| YC2015F | YC2016F | 0.08 | 0.50 | 0.07 | 0.37 | 0.08 | 0.46 |
| YC2015F | YC2016F | 0.04 | 0.61 | 0.02 | 0.24 | 0.06 | 0.60 |
| YC2015N + YC2015F | YC2016F | 0.07 | 0.32 | 0.07 | 0.34 | 0.08 | 0.39 |
| YC2015N | YC2017 | 0.06 | 0.48 | 0.07 | 0.55 | 0.04 | 0.28 |
| YC2015F | YC2017 | 0.06 | 0.54 | 0.05 | 0.40 | 0.03 | 0.26 |
| YC2015N + YC2015F | YC2017 | 0.08 | 0.46 | 0.08 | 0.45 | 0.06 | 0.35 |
| YC2016N | YC2017 | 0.29 | 0.78 | 0.29 | 0.75 | 0.27 | 0.69 |
| YC2016F | YC2017 | 0.20 | 1.01 | 0.20 | 0.90 | 0.22 | 1.02 |
| YC2016N + YC2016F | YC2017 | 0.32 | 0.82 | 0.35 | 0.84 | 0.33 | 0.79 |
| YC2015N + YC2015F + YC2016N + YC2016F | YC2017 | 0.26 | 0.82 | 0.27 | 0.91 | 0.26 | 0.84 |
| YC2015N | YC2018 | 0.08 | 0.44 | 0.08 | 0.43 | 0.05 | 0.24 |
| YC2015N + YC2015F | YC2018 | 0.06 | 0.28 | 0.10 | 0.40 | 0.09 | 0.40 |
| YC2016N | YC2018 | 0.32 | 0.72 | 0.32 | 0.68 | 0.32 | 0.64 |
| YC2016F | YC2018 | 0.12 | 0.59 | 0.12 | 0.56 | 0.11 | 0.46 |
| YC2016N + YC2016F | YC2018 | 0.33 | 0.70 | 0.35 | 0.67 | 0.33 | 0.64 |
| YC2017 | YC2018 | 0.39 | 0.67 | 0.42 | 0.69 | 0.41 | 0.66 |
| YC2016N + YC2017 | YC2018 | 0.44 | 0.64 | 0.46 | 0.66 | 0.45 | 0.60 |
| YC2016F + YC2017 | YC2018 | 0.40 | 0.68 | 0.41 | 0.67 | 0.45 | 0.69 |
| YC2016N + YC2016F + YC2017 | YC2018 | 0.44 | 0.66 | 0.48 | 0.65 | 0.46 | 0.61 |
| YC2015N + YC2016N +YC2016F + YC2017 | YC2018 | 0.39 | 0.65 | 0.43 | 0.69 | 0.42 | 0.64 |
| YC2015F + YC2016N + YC2016F + YC2017 | YC2018 | 0.46 | 0.72 | 0.49 | 0.74 | 0.49 | 0.68 |
| YC2015N + YC2015F + YC2016N +YC2016F + YC2017 | YC2018 | 0.41 | 0.69 | 0.44 | 0.75 | 0.44 | 0.69 |

Table S5: Correlation of minor allele frequencies between populations

|  | YC2015F | YC2016N | YC2016F | YC2017 | YC2018 |
| --- | --- | --- | --- | --- | --- |
| YC2015N | 0.49 | 0.68 | 0.62 | 0.62 | 0.68 |
| YC2015F |  | 0.52 | 0.69 | 0.54 | 0.51 |
| YC2016N |  |  | 0.63 | 0.69 | 0.61 |
| YC2016F |  |  |  | 0.61 | 0.58 |
| YC2017 |  |  |  |  | 0.74 |
